# Cohort Profile: The Vukuzazi (‘Wake Up and Know Yourself’ in isiZulu) population science programme

**DOI:** 10.1093/ije/dyab229

**Published:** 2021-11-29

**Authors:** Resign Gunda, Olivier Koole, Dickman Gareta, Stephen Olivier, Ashmika Surujdeen, Theresa Smit, Tshwaraganang Modise, Jaco Dreyer, Gregory Ording-Jespersen, Day Munatsi, Siyabonga Nxumalo, Thandeka Khoza, Ngcebo Mhlongo, Kathy Baisley, Janet Seeley, Alison D Grant, Kobus Herbst, Thumbi Ndung'u, Willem A Hanekom, Mark J Siedner, Deenan Pillay, Emily B Wong, the Vukuzazi Study Team

**Affiliations:** Africa Health Research Institute, KwaZulu-Natal, South Africa; Division of Infection and Immunity, University College London, London, UK; School of Nursing and Public Health, University of KwaZulu-Natal, Durban, South Africa; Africa Health Research Institute, KwaZulu-Natal, South Africa; London School of Hygiene & Tropical Medicine, London, UK; Africa Health Research Institute, KwaZulu-Natal, South Africa; Africa Health Research Institute, KwaZulu-Natal, South Africa; Africa Health Research Institute, KwaZulu-Natal, South Africa; Africa Health Research Institute, KwaZulu-Natal, South Africa; Africa Health Research Institute, KwaZulu-Natal, South Africa; Africa Health Research Institute, KwaZulu-Natal, South Africa; Africa Health Research Institute, KwaZulu-Natal, South Africa; Africa Health Research Institute, KwaZulu-Natal, South Africa; Africa Health Research Institute, KwaZulu-Natal, South Africa; Africa Health Research Institute, KwaZulu-Natal, South Africa; Africa Health Research Institute, KwaZulu-Natal, South Africa; Africa Health Research Institute, KwaZulu-Natal, South Africa; London School of Hygiene & Tropical Medicine, London, UK; Africa Health Research Institute, KwaZulu-Natal, South Africa; London School of Hygiene & Tropical Medicine, London, UK; Africa Health Research Institute, KwaZulu-Natal, South Africa; London School of Hygiene & Tropical Medicine, London, UK; School of Laboratory Medicine and Medical Sciences, University of KwaZulu-Natal, Durban, South Africa; School of Clinical Medicine, College of Health Sciences, University of KwaZulu-Natal, Durban, KwaZulu-Natal, South Africa; Africa Health Research Institute, KwaZulu-Natal, South Africa; DSI-MRC South African Population Research Infrastructure Network, South African Medical Research Council, Durban, South Africa; Africa Health Research Institute, KwaZulu-Natal, South Africa; Division of Infection and Immunity, University College London, London, UK; HIV Pathogenesis Programme, Doris Duke Medical Research Institute, Nelson R. Mandela School of Medicine, University of KwaZulu-Natal, Durban, South Africa; Ragon Institute of MGH, MIT, and Harvard, Harvard Medical School, Cambridge, MA, USA; Max Planck Institute for Infection Biology, Berlin, Germany; Africa Health Research Institute, KwaZulu-Natal, South Africa; Division of Infection and Immunity, University College London, London, UK; Africa Health Research Institute, KwaZulu-Natal, South Africa; School of Clinical Medicine, College of Health Sciences, University of KwaZulu-Natal, Durban, KwaZulu-Natal, South Africa; Division of Infectious Diseases, Massachusetts General Hospital, Boston, MA, USA; Department of Medicine, Harvard Medical School, Boston, MA, USA; Africa Health Research Institute, KwaZulu-Natal, South Africa; Division of Infection and Immunity, University College London, London, UK; Africa Health Research Institute, KwaZulu-Natal, South Africa; Division of Infection and Immunity, University College London, London, UK; Division of Infectious Diseases, Massachusetts General Hospital, Boston, MA, USA; Division of Infectious Diseases, Department of Medicine, University of Alabama at Birmingham, Birmingham, AL, USA

Key Features

- The Vukuzazi programme was established to address public health responses and scientific priorities in light of the convergence of the HIV, tuberculosis and non-communicable disease epidemics in South Africa.
- The programme is a nested closed cohort of the Population Intervention Programme and performed community-based health phenotyping in the uMkhanyakude district of rural KwaZulu-Natal, South Africa, while simultaneously creating a data, image and bio-sample repository.
- Baseline data collection began in May 2018 and was completed in March 2020, having enrolled 50% of the eligible population aged 15 years and over [18 041: 12 230 female (68%) female and 5811 male (32%)].
- The community-based phenotyping consisted of: a nurse-administered health questionnaire on personal history of HIV, tuberculosis, diabetes, hypertension, cancer, tobacco and alcohol use; quality of life; anthropometric measurements and blood pressure readings; venous blood sample for clinical testing and bio-banking; digital chest radiograph, sputum collection and sputum mycobacterial tests; as well as rectal swab collection for biobanking.
- Data for secondary analysis, access to the biological samples and imaging data are available through request on this link: [https://data.ahri.org].

## Why was the cohort set up?

In 2000, the Africa Centre Demographic Information System (ACDIS),^1^ a demographic surveillance platform, was established in the uMkhanyakude district of rural KwaZulu-Natal, one of the world's most intensely HIV-affected regions. Due to massive public health efforts that have increased access to antiretroviral therapy in this population over the past 15 years, HIV-associated mortality is declining and life expectancy is rising.^2^ Tuberculosis (TB) has overtaken HIV as the leading cause of mortality in South Africa,^3^ and non-communicable disease (NCD) morbidity and mortality are rising nationally^4^ and across sub-Saharan Africa.^5^ In response to the changing scientific and public health priorities posed by the intersection of the HIV, TB and NCDs epidemics, the Africa Health Research Institute (AHRI) expanded the Africa Centre Demographic Information System to form the Population Intervention Programme (PIP) in 2017.^6^ In 2018, Vukuzazi (‘Wake up and know yourself’ in isiZulu) was established as a nested closed cohort of PIP. Vukuzazi offered community-based health phenotyping and comprehensive bio-sampling to all resident adult (≥15 years) members of the southern part of the PIP study area, while building upon the existing wealth of demographic and HIV information collected over the previous 20 years. The objective of the programme was to determine the prevalence and overlap of infectious diseases and NCDs in the population 20 years into the HIV epidemic. Additionally, the programme aimed to create a data, image and bio-sample repository that could be used to understand the host, pathogen, social and environmental determinants of specific states of health and disease in the population, and provide the basis for follow-up intervention studies.

**Figure 1:**
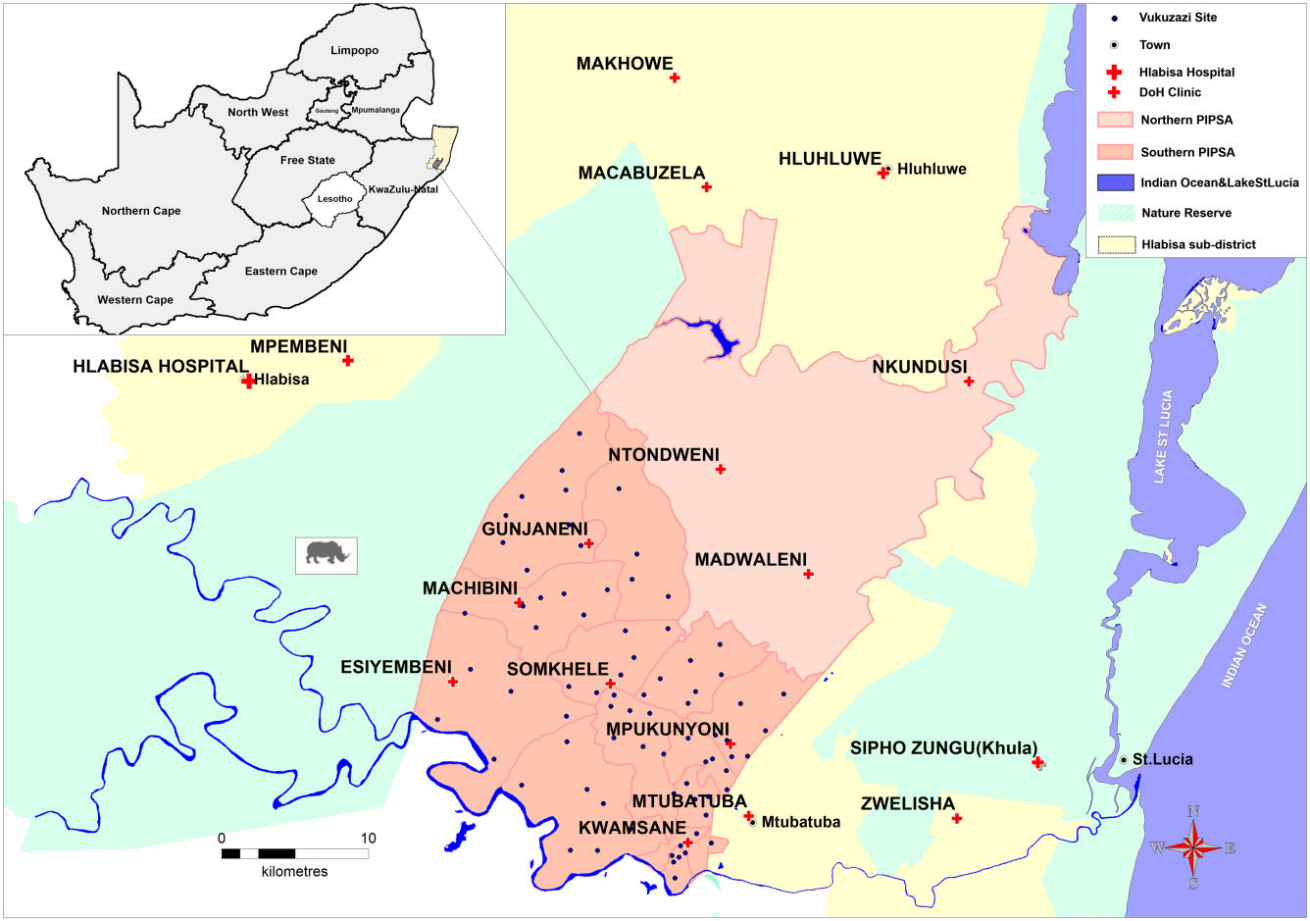
Location of the study area, showing the Vukuzazi sites within the demographic surveillance area

The Vukuzazi programme was conducted in the southern part of the PIP area,^6^**(**Figure 1**)** which corresponds with the original Africa Centre Demographic Information System surveillance area, near the market town of Mtubatuba. The area covers 438 km^2^ and includes a population of approximately 12 000 households with 65 000 residents (people who spend most of their nights in the surveillance area) and 30 000 non-residents (people who are household members but spend most of their nights outside the surveillance area). About ∼40% of resident members are younger than 15 years. Households in the southern PIP have been under continuous demographic surveillance since 2000.^1^ In 2003, annual HIV serosurveys were added to describe the demographic social and health impacts of the rapidly progressing HIV epidemic.^1^ The PIP area has 11 peripheral primary health care clinics (of which seven are in the southern part), and one district hospital lies outside the study area. The location of the area covered by the Vukuzazi programme, sites where the mobile camp enrolled participants and other features of the area are shown in Figure 1.

The scientific objectives and proposed activities of the Vukuzazi programme were conceptualized and developed in consultation with the AHRI Community Advisory Board (CAB) and other local stakeholders, including elected and traditional leaders and representatives of the local Department of Health (DoH).

## Who is in the cohort?

Ethical approval for the study was obtained from the Ethics Committees of the University of KwaZulu-Natal (BE560/17), London School of Hygiene & Tropical Medicine (#14722), the Partners Institutional Review Board (2018P001802), and from the University of Alabama at Birmingham (#300007237).

The eligible study population consisted of all resident members of the southern PIP area who were aged ≥15 years during the baseline data collection (May 2018–March 2020). Non-resident members were excluded from participation.

**Figure 2:**
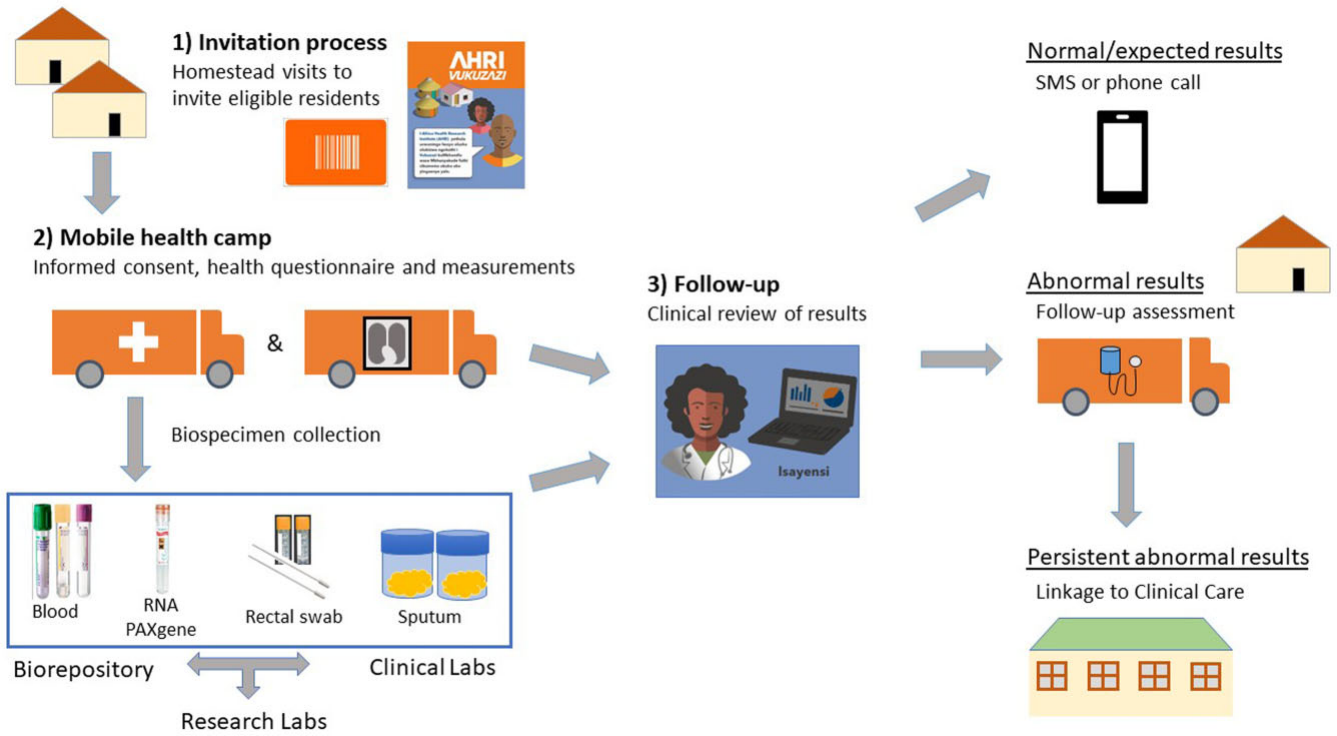
Components of the Vukuzazi programme

**Figure 3:**
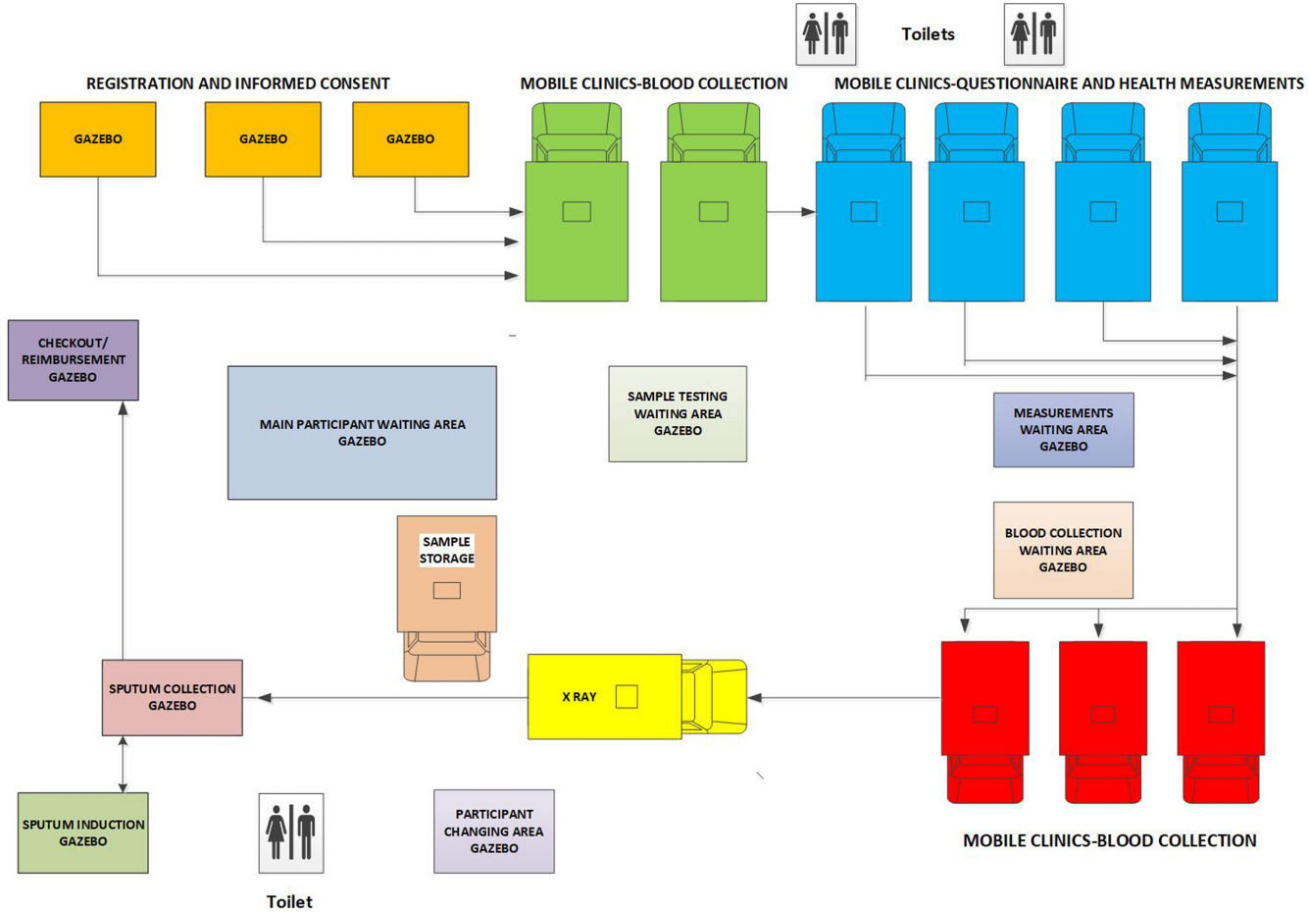
The Vukuzazi mobile health camp

Participants were recruited in a two-stage process (Figure 2): (i) a household visit during which all eligible participants were invited to participate (the invitation process); and (ii) a formal informed consent and enrolment process at the Vukuzazi health camp (Figure 3).

### Invitation process at the participant’s homestead

This component was scheduled approximately 1 week before the Vukuzazi mobile health camp visited an area. We used the demographic surveillance data to populate an eligibility list, compiled by fieldworkers at the most recent household survey but no more than 1 year before the Vukuzazi visit. Based on this list, the community-based study team visited the household and invited all eligible members to the Vukuzazi health camp. The team explained the rationale for the study, using an information brochure written in isiZulu which was left with the household. Additionally, the team left personalized invitation cards for each eligible member which included the individual’s unique PIP identification number, a quick response (QR) barcode, appointment date and the location of the Vukuzazi mobile health camp. If no residents were home when a household was visited, three visit attempts were conducted before a household was considered non-contactable.

### Informed consent and enrolment at the Vukuzazi mobile health camp

During the 22-month study data collection period, the Vukuzazi mobile health camp moved through the PIP area with the goal of conducting procedures within two kilometres of all eligible homesteads. Vukuzazi camp sites were selected based on accessibility, sufficient space and mobile connectivity. Permission was sought from the local traditional authorities and leaders before selection of the final sites. The mobile health camp stayed on average 2 days at each location. In total, the camp was set up at 78 sites over the observation period (Figure 1). Participants presented to the health camp with their unique invitation card. After verification of their identity, they proceeded to the next station for the informed consent process and an explanation of the study components, including the risks and benefits of participation, as well as information about storage of samples for genetic analyses and future research. Those who consented were given a barcoded wristband which functioned as a unique identifier during their Vukuzazi camp interaction, checking their identifiers at each station.

#### Recruitment

**Figure 4:**
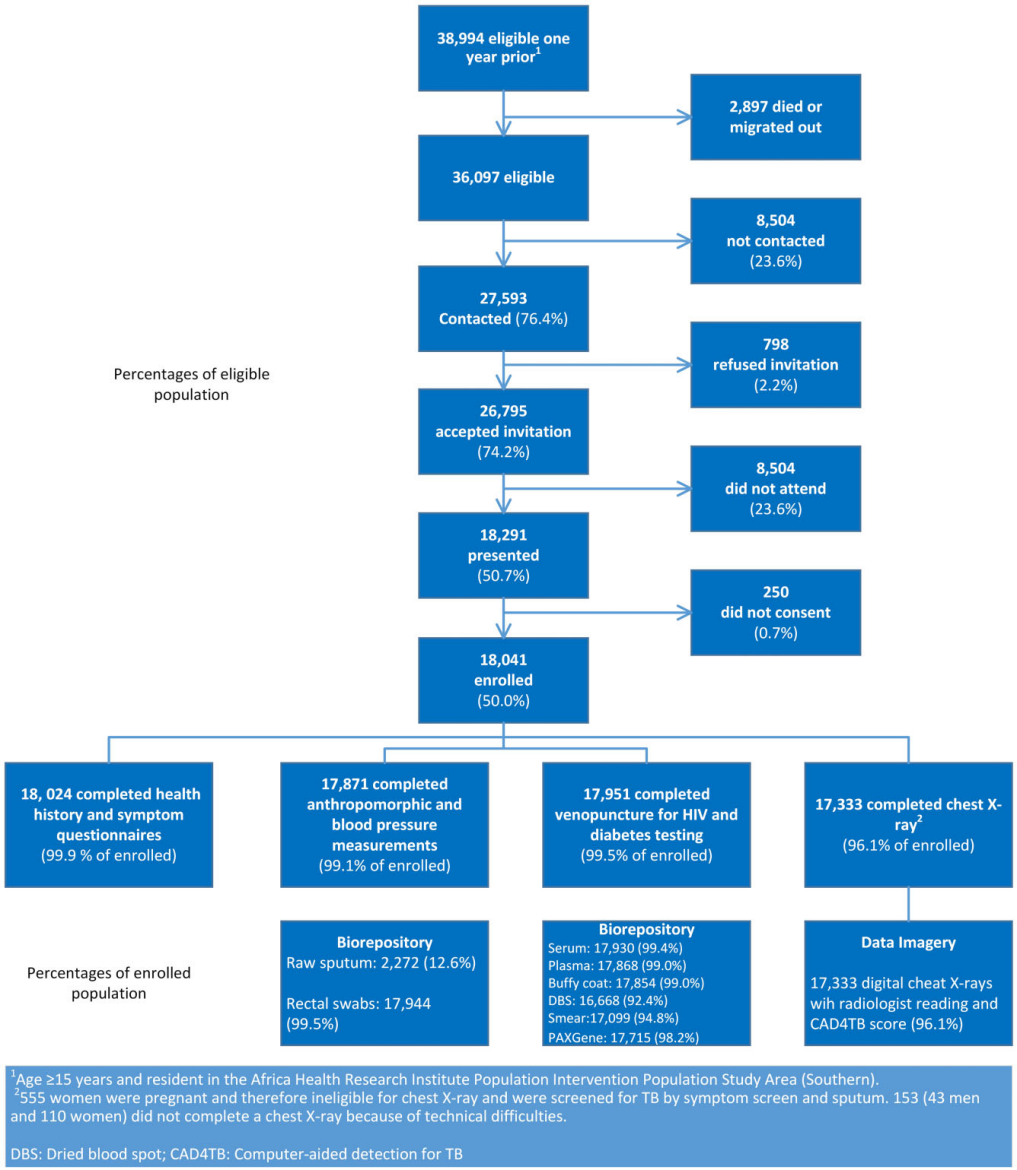
Recruitment and participation in the Vukuzazi programme

About 39 000 individuals were eligible 1 year before data collection. At the moment of data collection, about 3000 of them had died or moved out of the study area (Figure 4). This resulted in 36 097 individuals eligible for enrolment, of whom approximately three-quarters were contacted and accepted the invitation to visit the Vukuzazi mobile camp (about one-quarter were unable to be contacted, and of the contacted individuals only 2% refused the invitation). Among the 26 795 individuals who accepted the invitation, however, approximately one-quarter of eligible participants did not appear at the Vukuzazi camp, resulting in 18 041 individuals (50.0% of the eligible population) who enrolled in the Vukuzazi programme.

**Figure 5:**
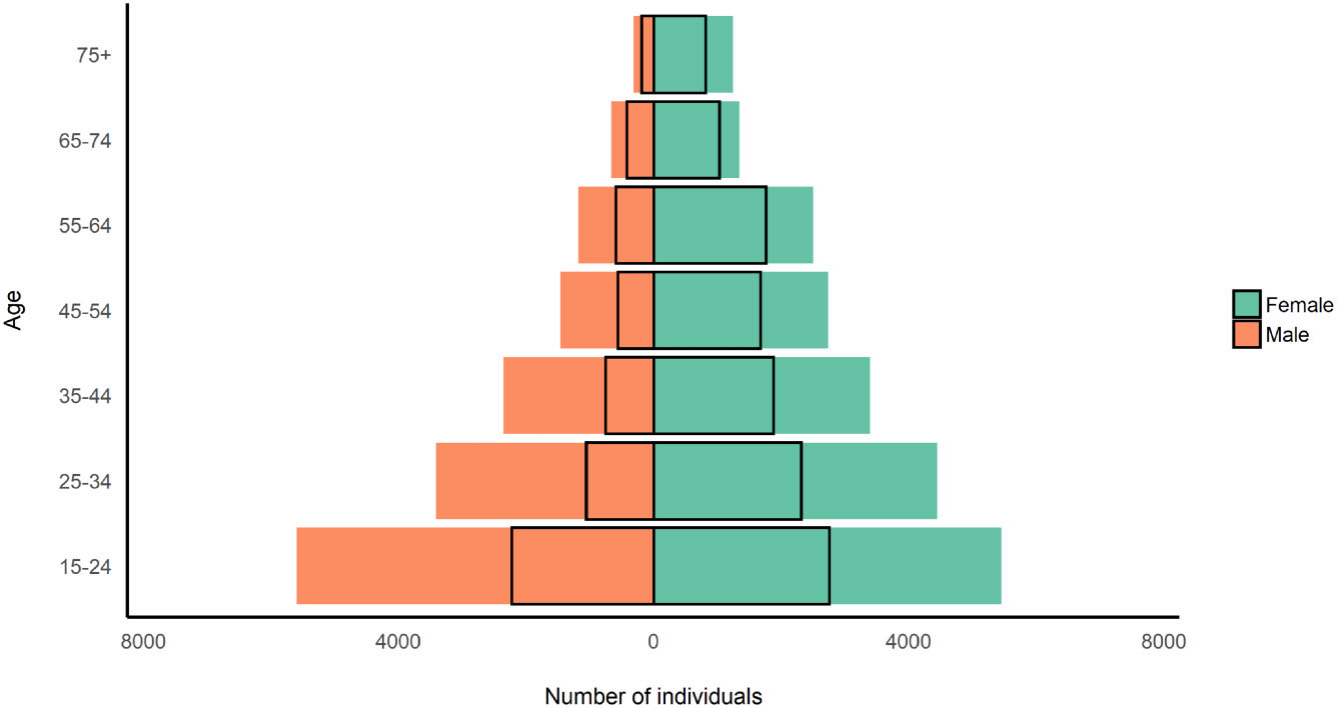
Population pyramid of the eligible population and Vukuzazi participants (at time of invitation or recruitment)

**Figure 6:**
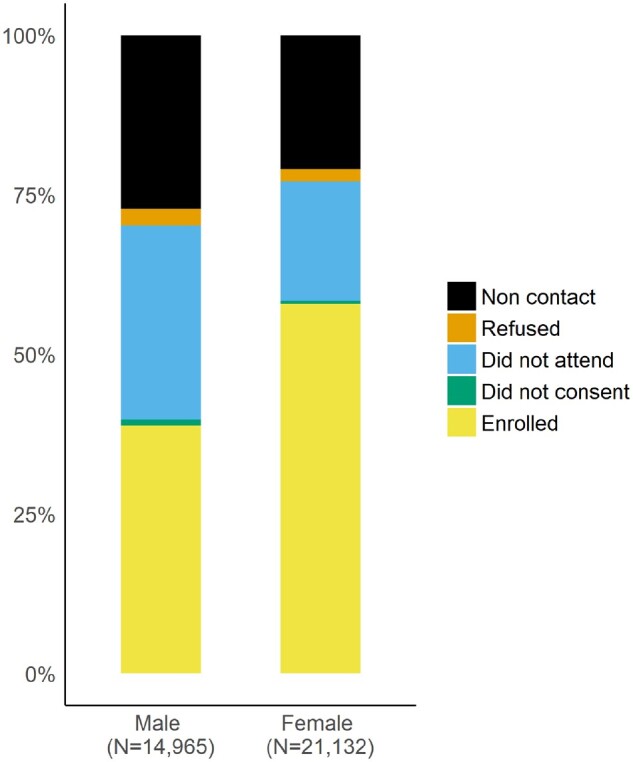
Contact and Vukuzazi health camp attendance rates by sex for eligible members of the demographic surveillance population

The Vukuzazi programme enrolled participants across the age range of the eligible population, with slight under-representation of the younger ages (Table 1). Females outnumbered males in the underlying population structure (Figure 5) and enrolled in higher proportions than males (Figure 6). Most participants resided in a rural (64%) or peri-urban (31%) area of the study area and had very high rates of unemployment (57%, Table 1). They were less highly educated, had lower rates of employment, were less likely to have out-migrated during the past 5 years and had a lower socioeconomic score compared with eligible non-participants. They were also more likely to have visited a clinic in the past year and to have ever consented to home-based HIV counselling and testing during the individual surveys (Table 1).

**Table 1:** Demographic characteristics of Vukuzazi participants, non-participants and eligible population

Characteristic	Vukuzazi participants	Non-participants	Eligible population	*P*-value^b^
	*N *= 18 041^a^	*N* = 18 056^a^	*N* = 36 097^a^	
Age group				<0.001
15–24	4977 (28%)	6072 (34%)	11 049 (31%)	
25–34	3371 (19%)	4483 (25%)	7854 (22%)	
35–44	2634 (15%)	3119 (17%)	5753 (16%)	
45–54	2240 (12%)	1952 (11%)	4192 (12%)	
55–64	2353 (13%)	1320 (7%)	3673 (10%)	
65+	2466 (14%)	1110 (6%)	3576 (10%)	
Sex				<0.001
Male	5811 (32%)	9154 (51%)	14 965 (41%)	
Female	12 230 (68%)	8902 (49%)	21 132 (59%)	
Education				<0.001
None	4532 (25%)	3855 (22%)	8387 (24%)	
Less than secondary	6737 (38%)	5173 (29%)	11 910 (33%)	
Secondary and above	6666 (37%)	8719 (49%)	15 385 (43%)	
Missing	106	309	415	
Marital status				<0.001
Single	3757 (24%)	4015 (27%)	7772 (26%)	
Married/living as married	9647 (62%)	9761 (66%)	19 408 (64%)	
Separated/divorced/widowed	2155 (14%)	1042 (7%)	3197 (11%)	
Missing	2482	3238	5720	
Employment status^c^				<0.001
Unemployed	3887 (57%)	3420 (37%)	7307 (45%)	
Employed^d^	2979 (43%)	5921 (63%)	8900 (55%)	
Missing	11 175	8715	19 890	
Residence location				<0.001
Urban	950 (5%)	1899 (11%)	2849 (8%)	
Peri-urban	5599 (31%)	6214 (35%)	11 813 (33%)	
Rural	11 436 (64%)	9863 (55%)	21 299 (59%)	
Missing	56	80	136	
Socioeconomic status^e^				<0.001
Lowest	2118 (12%)	1606 (9.4%)	3724 (11%)	
Low	4712 (27%)	3727 (22%)	8439 (24%)	
Middle	4216 (24%)	3716 (22%)	7932 (23%)	
High	3115 (18%)	3122 (18%)	6237 (18%)	
Highest	3307 (19%)	4833 (28%)	8140 (24%)	
Missing	573	1052	1625	
Out-migration during the past 5 years	2315 (13%)	3714 (21%)	6029 (17%)	<0.001
Any clinic visit in the past year (ClinicLink)	9561 (53%)	5940 (33%)	15 501 (43%)	<0.001
Ever consented for HIV testing during the individual surveys?	7389 (41%)	3904 (22%)	11 293 (31%)	<0.001

a Statistics presented: *n* (%).

b Statistical tests performed: chi square test of independence.

c Employment status calculated among members of the resident population in the labour force.

d Department of Statistics South Africa's strict definition of unemployment.

e Socioeconomic status: an asset-based index created using principal components analysis (PCA) on a standard list of questions about household items, water source, toilet type and electricity source.

## How often have they been followed up?

Vukuzazi is nested within the AHRI PIP,^6^ which will allow for both demographic and health system follow-up of participants. Participants will be observed prospectively three times per year through the PIP demographic surveillance activities, which will record changes to their household residence, demographic and vital status. In addition, participants’ prospective clinical data will be collected through clinic and hospital data systems established through PIP.^6^^,^^7^ In 2017, AHRI implemented the ClinicLink system^6^^,^^7^ to collect the date of and self-reported reasons for all visits by individuals who attend one of the 11 clinics in the PIP area (whether self-referred or referred to care after screening tests). In this ClinicLink system, data are electronically captured and individuals are linked to their PIP identification number at the time of the visit. Data on all admissions to Hlabisa District Hospital (the local referral hospital) are collected through the Hospital Information System. Successful referrals or clinic attendance were monitored through ClinicLink. Successful referrals from Vukuzazi were entered into the relevant DoH care pathway by an AHRI professional nurse based at the health facility.

Finally, in accordance with ongoing PIP activities, verbal autopsy will be performed for all deaths occurring in the population.^6^ These follow-up mechanisms allow Vukuzazi’s 2018–20 data to serve as a baseline assessment of a longitudinal cohort that includes clinical outcome assessment. Additional nested disease- and health behaviour-specific protocols will provide additional specific follow-up information.^8^ Long-term plans for the cohort will be driven both by scientific priorities and funding; currently we are considering repeating clinical measures every 3 to 5 years.

## What has been measured?

The majority of measurements and samples were collected at the Vukuzazi mobile health camp; participants with abnormal measurements or results had a follow-up home visit with additional data collected at that time. The participant flow through the Vukuzazi mobile camp is summarized in Figure 3 and the data types collected during the two visits are described in Table 2. The mobile health camp consisted of module elements that could be used in parallel to accommodate up to 100 participants per day (Figure 3).

**Table 2:** Data collected at the Vukuzazi health camp, during the follow-up visit at home and through linkage with other systems

	Topic	Details	Who
**Vukuzazi Health Camp**			
Questionnaire	Health history	HIV, TB, cardiovascular disease, diabetes (WHO STEPS questionnaire)	All
	Symptoms	Cardinal TB symptoms, COPD symptoms	All
	Alcohol and tobacco use	WHO STEPS questionnaire	All
	Quality of life	EuroQOL EQ5D	All
Measurements	Anthropometry	Height, weight, BMI, MUAC, hip circumference, abdominal circumference	All
	Blood pressure	3 measurements (WHO STEPS protocol)	All
**Specimens**			
Urine specimen	Pregnancy	Urine beta-HCG	All women 15–49 years
Rectal swab	Microbiome	Storage for future testing	All
Blood specimens	HIV Ab/Ag		All
	Viral Load, CD4		Positive HIV Ab/Ag
	HbA1c		All
	Plasma, serum, dried blood spot, PAXGene, blood smear, buffy coat	Storage for future testing	All who consent to storage of specimens
Sputum	TB	GeneXpert Mtb/RIF and liquid mycobacterial culture	Pregnant women and individuals with CAD4TB score above threshold
		Culture and drug susceptibility testing	All
	Mycobacterium tuberculosis isolate	Storage for future testing	All with a positive liquid culture
	Decontaminated sputum	Storage for future testing	All
Imagery	Digital chest X-ray		All except for pregnant women
**Follow-up visit at home**			
Measurements	Blood pressure	3 measurements (WHO STEPS protocol)	Elevated blood pressure (≥140 or 90) and not known hypertensive during health camp visit
	Blood glucose	Fasting (if feasible)	Elevated HbA1c during health camp visit
**Linkage with other systems**			
Population Intervention Programme (PIP)	Household demographics	Household members (dates of birth, sex, relationship to household head, marital status, residency status), births, deaths, in- and out-migration	All households in PIP
	Household socioeconomic data	Household assets, Household infrastructure (water, sanitation, electricity), food security, experience of violence	All households in PIP
	Individual socioeconomic data	Education, employment	All individuals who are members of households in PIP
	Individual—HIV status	HIV status	All residents aged ≥15 years in PIP
		Self-reported: HIV status, when last tested negative/positive, currently on ART	
	Individual—sexual behaviour	Pregnancy history (women), contraceptive use (women), number of children fathered (men), sexual activity, attitudes to condom use	All residents aged ≥15 years in PIP
	Individual—general health	Self-reported: hospitalized in past year, hypertension diagnosis/treatment, diabetes diagnosis/treatment,	All residents aged ≥15 years in PIP
		TB diagnosis/treatment circumcised (men)	
ClinicLink	Clinic attendance	Date of clinic visit, self-reported reason for clinic attendance	Individuals attending one of 11 clinics serving the PIP surveillance area
Hospital Information System	Hospital admissions	Admission date, discharge date, ward admitted to, ICD-10 diagnosis, discharge status	All admissions to Hlabisa hospital except for routine deliveries
TIER.net	Electronic patient records for individuals on ART	Clinic visits for ART care, viral load, CD4 counts, ART regimen at initiation, changes in ART regimen	Individuals on ART in any of 17 clinics in the Hlabisa health sub-district and Hlabisa hospital
	Electronic patient records for individuals on TB treatment	Clinic visits for TB care, TB regimen	Individuals on TB treatment in any of 17 clinics in the Hlabisa health sub-district and Hlabisa hospital

TB, tuberculosis; WHO STEPS, World Health Organization STEPwise; COPD, chronic obstructive pulmonary disease; BMI, body mass index; MUAC, mid-upper arm circumference; urine beta-HCG, urine beta human chorionic gonadotropin; HIV Ab/Ag, HIV antibody/antigen; GeneXpert Mtb/RIF, GeneXpert Mycobacterium tuberculosis/Rifampin; CAD4TB, Computer-Aided Detection for TB; HbA1c, glycated haemoglobin; PIP, Population Intervention Programme; ICD-10, International Statistical Classification of Diseases and Related Health Problems 10th Revision; ART, antiretroviral therapy.

After completing the informed consent procedure, participants submitted two self-administered rectal swabs, and women of childbearing age submitted a urine sample to ascertain pregnancy status. An enrolled nurse then administered a health questionnaire (see Supplementary material, available as Supplementary data at *IJE* online) on personal history of HIV, tuberculosis, diabetes, hypertension, cancer, tobacco and alcohol use,^9^ symptoms of TB (cough, fever, night sweats of any duration or unintended weight loss),^10^ and quality of life.^11^

Next, a research nurse performed anthropometric measurements (Table 2) and blood pressure readings following the World Health Organization STEPwise protocol.^9^ After 30 min of inactivity, three blood pressure measurements, with 5 min resting time in between, were collected using portable electronic devices (OMRON Healthcare, Model M6). Participants then moved to the blood station where 38.5 ml of blood was collected. All participants except for pregnant women underwent a digital chest X-ray (Canon CXDI-NE), performed in a mobile X-ray unit also equipped with an artificial intelligence computer-aided detection for TB (CAD4TB) score system (Version 5), which detects lung field abnormalities in real time.^12^ Any participant with one of the four cardinal TB symptoms, or a CAD4TB score above a pre-defined threshold or who was pregnant, was asked to submit a sputum sample. After completion of the chest X-ray and/or sputum collection, the visit to the health camp was completed at the exit station, where data were checked for completeness, and participants received a food parcel and reimbursement voucher (of the value of R 100—equivalent to approximately USD$6).

All chest X-rays were interpreted by CAD4TBv5 and, within 14 days, underwent a standardized interpretation by an experienced radiologist. The study physician reviewed all data from questionnaires, anthropomorphic and blood pressure measurements and laboratory and radiological testing, and initiated appropriate clinical management.

Participants with normal or expected findings (e.g. known HIV-positive on antiretroviral therapy with a suppressed viral load) received an SMS informing them that their results were normal. Participants who shared a phone or who had invalid test results received a phone call to ensure that the correct participant was contacted. Participants with abnormal findings (elevated blood pressure in someone not on antihypertensive treatment, or an X-ray suggestive of TB) or testing results (new HIV diagnosis, positive GeneXpert Mycobacterium tuberculosis/Rifampin (Xpert for MTB), glycated haemoglobin (HbA1c) >6.5%) were booked for an appointment for a home-based follow-up visit by a study nurse.

At the follow-up visits, the following measurements were made (Table 2): three blood pressure measurements (performed as described above) for people with elevated blood pressure at the Vukuzazi health camp; and point-of-care fasting blood glucose for people with HbA1c >6.5%. On the basis of the Vukuzazi health camp results and, when appropriate, the repeated measurements, referrals were made for further care at one of the local primary health care clinics or hospital.

In addition to the data collected through the Vukuzazi health camp, the following data on Vukuzazi participants are available through linkage with other data systems (Table 2): detailed demographic data (including education, socioeconomic status, marital status), sexual behaviour, household composition, structure and familial relationships (including family trees), household location (including geospatial coordinates), self-reported hospital attendance and diagnosis data, verbal autopsy data collected through the PIP surveillance,^6^ TB- and antiretroviral therapy-related data through Three Interlinked Electronic Register (TIER.net)^13^ and Covid-19-related data through the Covid-19 Surveillance protocol.^8^

## What has it found?

The data generated from the Vukuzazi study will create a clinical research platform for future longitudinal studies of HIV, TB, NCDs and multimorbidity. Use of mobile clinics and mobile X-ray units for multi-disease health screening enabled the study to enrol people within reach of their place of residence, thereby increasing participation rates. The study demonstrated the usefulness of digital health tools (such as mobile data collection tablets, digital chest X-rays) in collecting clinical data without need for paper records, thereby minimizing data entry and transfer errors and challenges associated with paper-based data collection. A key advantage of electronic data collection is speed of accessibility of data from multiple sources. We were able to return test results and conduct clinical review of all participants, leading to linkage to care for participants with abnormal results. The combination of decentralized mobile data collection and efficient participant-friendly data systems resulted in high rates of successful completion of study components.

The focus on multimorbidity was also a departure from many previous biomedical studies in the region, which often have a limited scope related to HIV and/or tuberculosis. The broad range of conditions considered might have helped reduce stigma associated with participation in the study.^14^

### Acceptance of community health screening and of the biobank platform

A qualitative sub-study of participant and non-participant experiences in Vukuzazi found that most participants had a positive impression of the research and their participation, although they emphasized the health screening components over the research components and had limited understanding of genetics and biobanking.^14^ Non-participants largely cited scheduling conflicts as their reason for not participating.

### Prevalence of infectious and non-communicable disease

Among the four conditions assessed, HIV had the highest overall population prevalence (34.2%). Previously undiagnosed active TB was prevalent in 1.0%, lifetime TB in 21.8%, elevated blood pressure in 23.0% and elevated blood glucose in 8.5% of the population. Appropriate treatment and resulting control of disease was highest for HIV (76.3%) and lower for elevated blood pressure (40.0%), active tuberculosis (31.3%) and elevated blood glucose (6.9%).^15^ Distribution of the four diseases was heterogeneously distributed between sexes, across the life course and geospatially. Rates of multimorbidity (more than one of the two diseases) were high and increased with age. Among the disease risk factors assessed, obesity had the highest overall prevalence (30.1%).

### Health-related quality of life

The contrast between controlled HIV and uncontrolled non-communicable diseases also appeared to have effects on health-related quality of life. The presence of controlled HIV was unexpectedly associated with better overall self-reported health (manuscript under review). This was in contrast to having more non-infectious conditions (whether controlled or not), which was associated with worse overall self-reported health. In South Africa and similar settings, where intersecting non-communicable and infectious disease epidemics are prevalent, incorporating multimorbidity into health care strategies and improving the integration of care may improve overall well-being.

### Asymptomatic TB

During the baseline survey we found that 1.0% of the screened population had microbiologically proven TB, and that 80% of these individuals were asymptomatic. Individuals newly diagnosed with active TB in community-based screening have fewer symptoms, compared with people diagnosed at health facilities (manuscript under review). Asymptomatic TB eludes traditional symptom-based TB screening algorithms, but in most cases it is identifiable by chest X-ray abnormality.^16^ In addition to often being asymptomatic, TB detected through community-based screening may have its own spectrum of radiological features compared with TB diagnosed in clinics and hospitals, making it a distinct use case for automated imaging algorithms.

### Screening for hypertension and diabetes

We found low positive predictive values of single screening measurements of blood pressure and blood glucose to detect hypertension and diabetes when compared with sequential testing on separate days. Single measurement screening for hypertension and diabetes resulted in approximately a 50% rate of potentially unnecessary or premature referrals into the health sector for these conditions (manuscript under review). These findings suggest that repeated testing for NCDs should be considered during community-based screenings, particularly in settings when there is concern for over-burdening health systems with potentially unnecessary referrals.

## What are the main strength and weaknesses?

### Strengths

The Vukuzazi programme has demonstrated that demographic surveillance programmes can be effectively used to support multi-disease health characterization of populations within a demographic surveillance area. The PIP surveillance provided a comprehensive sampling frame, enabling us to determine and quantify participation and disease prevalence rates. Data collected in Vukuzazi can be linked to previous and prospective demographic, socioeconomic, health and behavioural data routinely collected as part of the annual surveillance. The large size and completeness of the linked data, image and bio-sample repositories create the possibility for further detailed studies into the biological and social determinants of health and disease in the population, and interventions to improve outcomes.

### Weaknesses

We only enrolled half of the eligible participants. Contact rates were low among participants who were unavailable due to work, school and other social commitments. Various strategies such as varying recruitment times and working on weekends were employed, without significantly improving enrolment rates of difficult-to-enrol demographic sub-populations (e.g. working men). Non-participation could be non-random which needs to be considered in the interpretation of disease prevalence estimates. However, the existing wealth of information that has been collected on the eligible population since 2000 allows us to examine the difference between Vukuzazi participants and non-participants using statistical techniques to take into account non-response. This can be explored in future analyses and follow-up studies.

## Can I get hold of the data? And the biological samples or imaging data? Where can I find out more?

Researchers who would like to use the data for secondary analyses, or use the biological samples or imaging data, must complete a letter of intent accessible on this link: [https://data.ahri.org/index.php/home], in which they express their interest and outline the intended use of the data or biological samples. The Vukuzazi Scientific Steering Committee will review requests on an ongoing basis and invite proposals for collaboration. Vukuzazi data are accessible through the AHRI Data Repository after approval and completion of a data use agreement. Data documentation including questionnaires used for data collection and a full data dictionary are available on the AHRI Data Repository.

## Supplementary Data

Supplementary data are available at *IJE* online

## Funding

This research was funded in part, by the Wellcome Strategic Core award: 201433/Z/16/A, in part by the Bill and Melinda Gates Foundation (OPP175182) and in part by the Africa Health Research Institute. For the purpose of open access, the author has applied a CC BY public copyright license to any Author Accepted Manuscript version arising from this submission.

## Supplementary Material

dyab229_Supplementary_Data
