# Supplementary material for: Cohort Profile: The Vukuzazi (‘Wake Up and Know Yourself’ in isiZulu) population science programme
Source: Int J Epidemiol. 2021 Nov 29;51(3):e131–42. doi: 10.1093/ije/dyab229 (PMC9189966; doi:10.1093/ije/dyab229)
Supplement: dyab229_Supplementary_Data [file dyab229_supplementary_data.zip › ije-2021-02-0222-File009.pdf]

Supplementary Material – Questionnaire
Cohort Profile: The Vukuzazi ("Wake Up and Know Yourself" in isiZulu) Population Science Programme.

1) History of NCDs

History of Raised Blood Pressure		
**Question**	**Response**	**Code**
Have you ever had your blood pressure measured by a doctor or other health worker?	Yes 1<br>No 2 *If No, go to H4*	H1
Have you ever been told by a doctor or other health worker that you have raised blood pressure or hypertension?	Yes 1<br>No 2 *If No, go to H4*	H2a
Have you been told in the past 12 months?	Yes 1<br>No 2	H2b
In the past 6 months, have you seen a doctor or other health care worker about your high blood pressure?	Yes 1<br>No 2	H2c
In the past two weeks, have you taken any drugs (medication) for raised blood pressure prescribed by a doctor or other health worker?	Yes 1<br>No 2	H3
Have you ever seen a traditional healer for raised blood pressure or hypertension?	Yes 1<br>No 2 *If No, go to H6*	H4
Are you currently taking any herbal or traditional remedy for your raised blood pressure?	Yes 1<br>No 2	H5

History of Diabetes		
Have you ever had your blood sugar measured by a doctor or other health worker?	Yes 1<br>No 2 *If No, go to H10*	H6
Have you ever been told by a doctor or other health worker that you have raised blood sugar or diabetes?	Yes 1<br>No 2 *If No, go to H12*	H7a
Have you been told in the past 12 months?	Yes 1<br>No 2	H7b

In the past 6 months, have you seen a doctor or other health care worker about your raised blood sugar or diabetes?	Yes 1<br>No 2	H7c
In the past two weeks, have you taken any drugs (medication) for diabetes prescribed by a doctor or other health worker?	Yes 1<br>No 2	H8
Are you currently taking insulin for diabetes prescribed by a doctor or other health worker?	Yes 1<br>No 2	H9
Have you ever seen a traditional healer for diabetes or raised blood sugar?	Yes 1<br>No 2 *If No, go to H12*	H10
Are you currently taking any herbal or traditional remedy for your diabetes?	Yes 1<br>No 2	H11

**History of Raised Total Cholesterol**		
**Question**	**Response**	**Code**
Have you ever had your cholesterol (fat levels in your blood) measured by a doctor or other health worker?	Yes 1<br>No 2 *If No, go to H15*	H12
Have you ever been told by a doctor or other health worker that you have raised cholesterol?	Yes 1<br>No 2 *If No, go to H15*	H13a
Have you been told in the past 12 months?	Yes 1<br>No 2	H13b
In the past two weeks, have you taken any oral treatment (medication) for raised total cholesterol prescribed by a doctor or other health worker?	Yes 1<br>No 2	H14
Have you ever seen a traditional healer for raised cholesterol?	Yes 1<br>No 2 *If No, go to H17*	H15
Are you currently taking any herbal or traditional remedy for your raised cholesterol?	Yes 1<br>No 2	H16

History of Cardiovascular Diseases			
Have you ever had a heart attack or chest pain from heart disease (angina)?	Yes 1<br>No 2		H17
Are you currently taking aspirin regularly to prevent or treat heart disease?	Yes 1<br>No 2		H18
Are you currently taking statins (Lovastatin/Simvastatin/Atorvastatin or any other statin) regularly to prevent or treat heart disease?	Yes 1<br>No 2		H19
Did your mother or father have a heart attack before the age of 60?	Yes 1<br>No 2<br>Unknown 3		H20
Have you ever had a stroke (cerebrovascular accident or incident)?	Yes 1<br>No 2		H21
Are you currently taking aspirin regularly to prevent a stroke?	Yes 1<br>No 2		H22
Are you currently taking statins (Lovastatin/Simvastatin/Atorvastatin or any other statin) regularly to prevent or treat a stroke?	Yes 1<br>No 2		H23
Did your mother or father have a stroke before the age of 60?	Yes 1<br>No 2<br>Unknown 3		H24
**History of cancer**			
Have you been treated for cancer before?	Yes	1	CA1
	No	2	
	Unknown	3	
If yes, which cancer	Free text		CA2

**Quality of Life**<br>We will now ask you some questions about your general health.w<br>For the following questions, please indicate which statements best describe your own health state <u>today</u>.			
First, I would like to ask you about <u>mobility</u>.<br>Would you say you have …	No problems walking about<br>Some problems walking about<br>You are confined to bed	1<br>2<br>3	Q1
Next, I would like to ask you about <u>self-care</u>.<br>Would you say you have …	No problems with self-care<br>Some problems washing or dressing yourself<br>You are unable to wash or dress yourself	1<br>2<br>3	Q2
Next, I would like to ask you about your <u>usual activities</u>, for example work, study, housework, family or leisure activities.<br>Would you say you have …	No problems with performing your usual activities<br>Some problems with performing your usual activities<br>You are unable to perform your usual activities	1<br>2<br>3	Q3
Next, I would like to ask you about <u>pain or discomfort</u>.<br>Would you say you have …	No pain or discomfort<br>Moderate pain or discomfort<br>Extreme pain or discomfort	1<br>2<br>3	Q4
Finally, I would like to ask you about <u>anxiety or depression</u>.<br>Would you say you have …	Not anxious or depressed<br>Moderately anxious or depressed<br>Extremely anxious or depressed	1<br>2<br>3	Q5

I would now like to ask you to do a different task.<br>To help you say how good or bad your health state is, I would like you to try to picture in your mind a scale that looks a bit like a thermometer. Can you do that? The best health state you can imagine is marked 100 (one hundred) at the top of the scale and the worst state you can imagine is marked 0 (zero) at the bottom.<br><br>I would now like you to tell me the point on this scale where you would put your own health state today.	Integer (limited to 0-100)	Q6

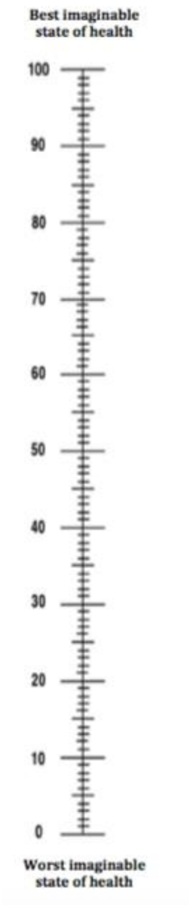

2) Behavioral measurements

Tobacco Use		
I am going to ask you some questions about tobacco use.		
**Question**	**Response**	**Code**
Do you **currently** smoke any **tobacco** products, such as cigarettes, cigars or pipes?<br>*(USE SHOWCARD)*	Yes 1<br>No 2 *If No, go to T8*	T1
Do you currently smoke tobacco products **daily**?	Yes 1<br>No 2	T2
How old were you when you **first started** smoking?	Age (years)<br>Don't know └─┴─┘ *If Known, go to T5a/T5aw*	T3
Do you remember how long ago it was?<br>*(RECORD ONLY 1, NOT ALL 3)*<br>*Don't know*	In Years └─┴─┘ *If Known, go to T5a/T5aw*	T4a
	OR in Months └─┴─┘ *If Known, go to T5a/T5aw*	T4b
	OR in Weeks └─┴─┘	T4c
On average, **how many** of the following products do you smoke **each day/week?**<br>*(IF LESS THAN DAILY, RECORD WEEKLY)*<br>*(RECORD FOR EACH TYPE, USE SHOWCARD)*<br>*Don't Know*	DAILY↓ WEEKLY↓	
	Manufactured cigarettes └─┴─┴─┴─┘ └─┴─┴─┴─┘	T5a/T5aw
	Hand-rolled cigarettes └─┴─┴─┴─┘ └─┴─┴─┴─┘	T5b/T5bw
	Pipes full of tobacco └─┴─┴─┴─┘ └─┴─┴─┴─┘	T5c/T5cw
	Cigars, cheroots, cigarillos └─┴─┴─┴─┘ └─┴─┴─┴─┘	T5d/T5dw
	Number of Shisha sessions └─┴─┴─┴─┘ └─┴─┴─┴─┘	T5e/T5ew
	Other └─┴─┴─┴─┘ └─┴─┴─┴─┘<br>*If Other, go to T5other, else go to T6*	T5f/T5fw
	Other (please specify): └─┴─┴─┴─┴─┴─┘	T5other/ T5otherw

During the past 12 months, have you tried to **stop smoking**?	Yes 1<br>No 2	T6
During any visit to a doctor or other health worker in the past 12 months, were you advised to quit smoking tobacco?	Yes 1<br>No 2<br>No visit during the past 12 months 3	T7
In the past, did you **ever smoke** any tobacco products? *(USE SHOWCARD)*	Yes 1 *If No, go to A1*<br>No 2 *If No, go to A1*	T8
In the past, did you **ever** smoke **daily**?	Yes 1<br>No 2	T9
How old were you when you **first started** smoking?	Age (years)<br>Don't know └─┴─┘ *If Known, go to T5a/T5aw*	T10
Do you remember how long ago it was?<br>*Don't know*	In Years └─┴─┘ *If Known, go to T5a/T5aw*<br>OR in Months └─┴─┘ *If Known, go to T5a/T5aw*<br>OR in Weeks └─┴─┘	T11a<br>T11b<br>T11c
How old were you when you **last quit** smoking?<br>Do you remember how long ago it was?	Age (years) └─┴─┘ *If Known, go to T5a/T5aw*<br>Don't know<br>In Years └─┴─┘ *If Known, go to T5a/T5aw*<br>OR in Months └─┴─┘ *If Known, go to T5a/T5aw*<br>OR in Weeks └─┴─┘	T12<br><br>T13a<br>T13b<br>T13c

Alcohol Consumption

The next questions ask about the consumption of alcohol.

Question	Response	Code
Have you **ever** consumed any alcohol such as beer, wine, spirits or *[add other local examples]*? *(USE SHOWCARD OR SHOW EXAMPLES)*	Yes 1<br>No 2 *If No, go to TB1*	A1
Have you consumed any alcohol within the **past 12 months**?	Yes 1 *If Yes, go to A4*<br>No 2	A2

Have you stopped drinking due to health reasons, such as a negative impact on your health or on the advice of your doctor or other health worker?	Yes 1<br>No 2	A3
During the past 12 months, **how frequently** have you had at least one standard alcoholic drink?<br>*(READ RESPONSES, USE SHOWCARD)*	Daily 1<br>5-6 days per week 2<br>3-4 days per week 3<br>1-2 days per week 4<br>1-3 days per month 5<br>Less than once a month 6	A4
Have you consumed any alcohol within the **past 30 days**?	Yes 1<br>No 2 *If No, go to TB1*	A5
During the past 30 days, on how many **occasions** did you have at least one standard alcoholic drink?	Number<br>Don't know └─┴─┘	A6
During the past 30 days, when you drank alcohol, how many **standard drinks on average** did you have during one drinking occasion?<br>*(USE SHOWCARD)*	Number<br>Don't know └─┴─┘	A7
During the past 30 days, what was the **largest number** of standard drinks you had on a single occasion, counting all types of alcoholic drinks together?	Largest number<br>Don't Know └─┴─┘	A8
During the past 30 days, how many times did you have **six or more** standard drinks in a single drinking occasion?	Number of times<br>Don't Know └─┴─┘	A9

During each of the **past 7 days**, how many standard drinks did you have each day?<br>*(USE SHOWCARD)*<br>*Don't Know*	Monday └─┴─┘	A10a
	Tuesday └─┴─┘	A10b
	Wednesday └─┴─┘	A10c
	Thursday └─┴─┘	A10d
	Friday └─┴─┘	A10e
	Saturday └─┴─┘	A10f
	Sunday └─┴─┘	A10g

Alcohol Consumption, continued

I have just asked you about your consumption of alcohol during the past 7 days. The questions were about alcohol in general, while the next questions refer to your consumption of homebrewed alcohol, alcohol brought over the border/from another country, any alcohol not intended for drinking or other untaxed alcohol. Please only think about these types of alcohol when answering the next questions.

Question	Response	Code
During the **past 7 days**, did you consume any **homebrewed** alcohol, any alcohol **brought over the border/from another country**, any alcohol **not intended for drinking** or other **untaxed** alcohol?<br>*[AMEND ACCORDING TO LOCAL CONTEXT]*<br>*(USE SHOWCARD)*	Yes 1<br>No 2 *If No, go to TB1*	A11
On average, **how many standard drinks** of the following did you consume **during the past 7 days**?<br>*[INSERT COUNTRY-SPECIFIC EXAMPLES]*<br>*(USE SHOWCARD)*<br>*Don't Know*	Homebrewed spirits, e.g. moonshine └─┴─┘	A12a
	Homebrewed beer or wine, e.g. beer, palm or fruit wine └─┴─┘	A12b
	Alcohol brought over the border/from another country └─┴─┘	A12c
	Alcohol not intended for drinking, e.g. alcohol-based medicines, perfumes, after shaves └─┴─┘	A12d
	Other untaxed alcohol in the country └─┴─┘	A12e

3) TB screening and asthma/COPD questions

TB screening questions			
Are you **currently** on TB treatment?	Yes	1	TB1
	No	2 If no, go to TB3	
If yes, have you received injections as part of this TB treatment?	Yes	1	TB2
	No	2	
	Unknown	3	
If yes, when did you start your current treatment for TB?	Date		TB2a
Have you been on TB treatment **before**?	Yes	1	TB3
	No	2 *If No, go to TB6*	
	Unknown	3 *If Unknown, go to TB6*	
If yes, how many times have you been on TB treatment?	Number of times		TB4a
Do you remember how old you were when you had your FIRST diagnosis of TB?	Yes	1 *If Yes, go to TB4c*	TB4b
	No		
Age	Years		TB4c
Do you remember the exact date or year when you completed your last TB treatment?	Exact date		TB5
	Year only		
	I don't know		
Has anyone in your homestead (or residential plot), during your lifetime, **ever** had TB disease? *(Including those who might not have started TB treatment but were told by a clinician that they had TB disease)*	Yes	1	TB6
	No	2	
	Unknown	3	

Is anybody in your homestead (or residential plot) **currently** on TB treatment?	Yes	1	TB7
	No	2	
	Unknown	3	
Do you **currently** have a cough?	Yes	1	TB8
	No	2 *If No, go to TB11*	
If yes, how long have you had the cough	Number of weeks		TB9
If yes, do you cough up sputum?	Yes	1	TB10
	No	2	
Do you have a fever?	Yes	1	TB11
	No	2	
If yes, how long have you had a fever?	Number of weeks		TB12
Do you have drenching night sweats? *(Sweating at night that soaks your clothes or beddings)*	Yes	1	TB13
	No	2 *If No, go to TB15*	
If yes, how long have you had drenching night sweats?	Number of weeks		TB14
Have you lost weight in the last 6 months? *(Explain to the participant that this is unexplained or unintentional weight loss)*	Yes	1	TB15
	No	2 *If No, go to TB171*	
If yes, how much weight have you lost in the last 6 months?	Kg		TB16
Do you have any other symptoms apart from those already mentioned? *(Prompt for TB specific symptoms [fatigue, haemoptysis, chest pain, and lymphadenitis])*	Yes	1	TB17
	No	2 *If No, go to AS1*	
If yes, which symptom	Fatigue		TB18
	Haemoptysis		
	Chest pain		
	Lymphadenitis		
If yes, duration of the symptom	Number of weeks		TB19

**Asthma / COPD questions**		
Have you ever been told by a doctor or other health worker that you have asthma or COPD?	Yes 1<br>No 2 *If No, go to AS4*	AS1
Have you been told in the past 12 months?	Yes 1<br>No 2	AS2
In the past two weeks, have you taken any medication for asthma/COPD prescribed by a doctor or other health worker?	Yes 1<br>No 2	AS3
Do you have shortness of breath after exercise or physical activity?	Yes 1<br>No 2	AS4
Do you wake up at night because of difficulties in breathing/shortness of breath?	Yes 1<br>No 2	AS5
Do you experience wheezing at night? *(Wheezing explained as a whistling sound that is made while breathing)*	Yes 1<br>No 2	AS6

4) HIV questions

HIV questions			
Have you ever received a test result for HIV?	Yes	1	HI1
	No	2 *If No, go to Section 5*	
	Unknown	3 *If No, go to Section 5*	
Have you ever had a positive HIV test result?	Yes	1	HI2
	No	2	
	Unknown	3	
HIV-positive			
Do you remember the exact date, or year of your first positive test result?	Exact date		HI3
	Year only		
	I don't know		
Have you ever been on ART?	Yes	1 *If Yes, go to HI5*	HI4
	No	2 *If No, go to Section 5*	
	Unknown		
Are you currently receiving ART?	Yes	1	HI5
	No	2	
	Refused to say	3	
Do you remember the exact date, or year when you first started ART did you first start ART?	Exact date		HI6
	Year only		
	I don't know		

Have you ever stopped/defaulted from ART treatment? (*Defaulted explained as not taken treatment for more than 3 months – 90 days)*	Yes	1	HI7
	No	2	
	Unknown	3	
How many times	Times		HI8
HIV-negative			
When was your last negative test result?	Date	0	HI9

5) Any other medical problem for which you regularly see a doctor (or traditional healer) or for which you have been prescribed a medication?

**Medical problems**	
**Any other medical problem for which you regularly see a doctor (or traditional healer) or for which you have been prescribed a medication?**	
**Medical problem (select)**	**Date/Year started**
Hypertension	
Diabetes	
Cholesterol	
Heart attack or stroke (cardiovascular disease)	
Tuberculosis	
Asthma/COPD	
HIV	
Other	

**Concurrent medication**					
**Name medication**	**Indication (select)**	**Date/year started**	**Currently taking**	**Taking as needed**	**Not taking**
	Hypertension				
	Diabetes				
	Cholesterol				
	Heart attack or stroke (cardiovascular disease)				
	Tuberculosis				
	Asthma/COPD				
	HIV				
	Other				

6) Operational questions

Operational questions			
Time of last meal or non-water drink	Date and time		
For women only:			
Do you remember the exact date, or year when your last menstrual period was?	Exact date		
	Year only		
	I don't know		
	Never		
