# Supplementary material for: Cohort Profile: The Vukuzazi (‘Wake Up and Know Yourself’ in isiZulu) population science programme
Source: Int J Epidemiol. 2021 Nov 29;51(3):e131–42. doi: 10.1093/ije/dyab229 (PMC9189966; doi:10.1093/ije/dyab229)
Supplement: dyab229_Supplementary_Data [file dyab229_supplementary_data.zip › ije-2021-02-0222-File010.pdf]

Author contributions

OK, RG, DG, AS, TS, JD, GO-J, DM, KB, JS, ADG, KH, TN, WH, MJS, EBW and DP were responsible for the study conceptualization and design.

RG, DG, SO, TM, JD, GO-J, DM, SN and TK collected and curated the data.

SO and DC did the analysis.

OK, RG, DG, SO, TS, JD, NM, KB, JS, TN, WH, EBW and DP wrote the paper.

OK, RG, SO and EBW verified the underlying data, and EBW is the guarantor for the paper.

All authors critically reviewed and edited the manuscript. All authors had access to all the data reported in the study.

Supplementary Material – Author contributions
Cohort Profile: The Vukuzazi ("Wake Up and Know Yourself" in isiZulu) Population Science Programme.

Vukuzazi Team - Below is a list of staff that significantly contributed to the implementation and conduct of Vukuzazi.

*Contributing authors

Name	Affiliation	Role
*Deenan Pillay	See byline at beginning of paper	Principal Investigator (2017-2019)
*Willem Hanekom	See byline at beginning of paper	Principal Investigator (2019-present)
*Emily Wong	See byline at beginning of paper	Co-Principal Investigator
*Mark Siedner	See byline at beginning of paper	Co-Principal Investigator
*Olivier Koole	See byline at beginning of paper	Co-Principal Investigator (2017-2019)
*Thumbi Ndung'u	See byline at beginning of paper	Co-investigator
*Thandeka Khoza	See byline at beginning of paper	Co-investigator (2019-present)
*Kobus Herbst	See byline at beginning of paper	Co-investigator
*Kathy Baisley	See byline at beginning of paper	Co-investigator
*Janet Seeley	See byline at beginning of paper	Co-investigator
*Alison Grant	See byline at beginning of paper	Co-investigator
*Resign Gunda	See byline at beginning of paper	Programme Manager
*Ashmika Surujdeen	See byline at beginning of paper	Study Coordinator
*Theresa Smit	See byline at beginning of paper	Head: Diagnostic Research
*Dickman Gareta	See byline at beginning of paper	Head: Research Data Management
*Day Munatsi	See byline at beginning of paper	Head: Research Data Systems
*Ngcebo Mhlongo	See byline at beginning of paper	Study Physician
*Tshwaraganang Modise	See byline at beginning of paper	Research Data Manager
*Jaco Dreyer	See byline at beginning of paper	Senior Research Data Manager
*Siyabonga Nxumalo	See byline at beginning of paper	Research Data Manager
*Stephen Olivier	See byline at beginning of paper	Statistician
*Gregory Ording-Jespersen	See byline at beginning of paper	Laboratory Data Supervisor
Innocentia Mpofana	Africa Health Research Institute	Diagnostic Laboratory Manager
Khadija Khan	Africa Health Research Institute	Biorepository Manager
Zizile Sikhosana	Africa Health Research Institute	Somkhele Laboratory Supervisor
Sashen Moodley	Africa Health Research Institute	Microbiology Laboratory Supervisor
Hollis Shen	Africa Health Research Institute	Head: Exploratory Research Division
Philippa Mathews	Africa Health Research Institute	Clinical Governance
Nompilo Buthelezi	Africa Health Research Institute	Training Coordinator
Hlolisile Khumalo	Africa Health Research Institute	Nursing Manager
Sanah Bucibo	Africa Health Research Institute	Professional Nurse
Nozipho Mbonambi	Africa Health Research Institute	Professional Nurse
Hloniphile Ngubane	Africa Health Research Institute	Professional Nurse
Thokozani Simelane	Africa Health Research Institute	Professional Nurse
Khanyisani Buthelezi	Africa Health Research Institute	Professional Nurse
Sphiwe Ntuli	Africa Health Research Institute	Professional Nurse
Nombuyiselo Zondi	Africa Health Research Institute	Professional Nurse

Siboniso Nene	Africa Health Research Institute	Professional Nurse
Bongumenzi Ndlovu	Africa Health Research Institute	Enrolled Nurse
Talente Ntimbane	Africa Health Research Institute	Enrolled Nurse
Mbali Mbuyisa	Africa Health Research Institute	Enrolled Nurse
Xolani Mkhize	Africa Health Research Institute	Enrolled Nurse
Melusi Sibiya	Africa Health Research Institute	Enrolled Nurse
Ntombiyenkosi Ntombela	Africa Health Research Institute	Enrolled Nurse
Mandisi Dlamini	Africa Health Research Institute	Enrolled Nurse
Hlobisile Chonco	Africa Health Research Institute	Enrolled Nurse
Hlengiwe Dlamini	Africa Health Research Institute	Enrolled Nurse
Doctar Mlambo	Africa Health Research Institute	Enrolled Nurse
Nonhlanhla Mzimela	Africa Health Research Institute	Enrolled Nurse
Zinhle Buthelezi	Africa Health Research Institute	Enrolled Nurse
Zinhle Mthembu	Africa Health Research Institute	Enrolled Nurse
Thokozani Bhengu	Africa Health Research Institute	Enrolled Nurse
Sandile Mthembu	Africa Health Research Institute	Enrolled Nurse
Phumelele Mthethwa	Africa Health Research Institute	Enrolled Nurse
Zamashandu Mbatha	Africa Health Research Institute	Enrolled Nurse
Welcome Petros Mthembu	Africa Health Research Institute	Enrolled Nurse
Anele Mkhwanazi	Africa Health Research Institute	Clinical Research Assistant Supervisor
Mandlakayise Zikhali	Africa Health Research Institute	Clinical Research Assistant Supervisor
Phakamani Mkhwanazi	Africa Health Research Institute	Clinical Research Assistant
Ntombiyenhlanhla Mkhwanazi	Africa Health Research Institute	Clinical Research Assistant
Rose Myeni	Africa Health Research Institute	Clinical Research Assistant
Fezeka Mfeka	Africa Health Research Institute	Clinical Research Assistant
Hlobisile Gumede	Africa Health Research Institute	Clinical Research Assistant
Nonceba Mfeka	Africa Health Research Institute	Clinical Research Assistant
Ayanda Zungu	Africa Health Research Institute	Clinical Research Assistant
Nonhlanhla Mfekayi	Africa Health Research Institute	Clinical Research Assistant
Smangaliso Zulu	Africa Health Research Institute	Clinical Research Assistant
Mzamo Buthelezi	Africa Health Research Institute	Clinical Research Assistant
Senzeni Mkhwanazi	Africa Health Research Institute	Clinical Research Assistant
Mlungisi Dube	Africa Health Research Institute	Clinical Research Assistant
Hosea Kambonde	iMarketing Consultants	IT Systems Developer
Lindani Mthembu	Africa Health Research Institute	Information Technology Assistant
Seneme Mchunu	Africa Health Research Institute	Information Technology Assistant
Sibahle Gumbi	Africa Health Research Institute	Research Admin Assistant
Tumi Madolo	Africa Health Research Institute	Research Data Manager
Thengokwakhe Nkosi	Africa Health Research Institute	Driver
Sibusiso Mkhwanazi	Africa Health Research Institute	Driver
Sibusiso Nsibande	Africa Health Research Institute	Driver
Mpumelelo Steto	Africa Health Research Institute	Driver
Sibusiso Mhlongo	Africa Health Research Institute	Driver
Velile Vellem	Aurum Innova (Pty) Ltd	Driver
Pfarelo Tshivase	Aurum Innova (Pty) Ltd	Driver
Jabu Kwinda	Aurum Innova (Pty) Ltd	Driver

Bongani Magwaza	Africa Health Research Institute	General Worker
Siyabonga Nsibande	Africa Health Research Institute	General Worker
Skhumbuzo Mthombeni	Africa Health Research Institute	General Worker
Sphiwe Clement Mthembu	Africa Health Research Institute	General Worker
Antony Rapulana	Africa Health Research Institute	Laboratory Technologist
Jade Cousins	Africa Health Research Institute	Laboratory Technologist
Thabile Zondi	Africa Health Research Institute	Laboratory Technologist
Nagavelli Padayachi	Africa Health Research Institute	Laboratory Technologist
Freddy Mabetlela	Africa Health Research Institute	Laboratory Technologist
Simphiwe Ntshangase	Africa Health Research Institute	Laboratory Technician/LIMS Administrator
Nomfundo Luthuli	Africa Health Research Institute	Laboratory Technician
Sithembile Ngcobo	Africa Health Research Institute	Laboratory Technologist
Kayleen Brien	Africa Health Research Institute	Laboratory Technologist
Sizwe Ndlela	Africa Health Research Institute	Laboratory Technician
Nomfundo Ngema	Africa Health Research Institute	Laboratory Technician
Nokukhanya Ntshakala	Africa Health Research Institute	Laboratory Technician
Anupa Singh	Africa Health Research Institute	Laboratory Technician
Rochelle Singh	Africa Health Research Institute	Laboratory Technician
Logan Pillay	Africa Health Research Institute	Laboratory Technician
Kandaseelan Chetty	Africa Health Research Institute	Laboratory Technician
Ashentha Govender	Africa Health Research Institute	Laboratory Technician
Pamela Ramkalawon	Africa Health Research Institute	Laboratory Research Technician
Nondumiso Mabaso	Africa Health Research Institute	Laboratory Intern
Kimeshree Perumal	Africa Health Research Institute	Laboratory Intern
Senamile Makhari	Africa Health Research Institute	Biorepository Laboratory Technician
Nondumiso Khuluse	Africa Health Research Institute	Biorepository Laboratory Technician
Nondumiso Zitha	Africa Health Research Institute	Biorepository Research Assistant
Hlengiwe Khathi	Africa Health Research Institute	Biorepository Research Assistant
Mbuti Mofokeng	Africa Health Research Institute	Clinical Specimen Driver/Laboratory Assistant
Nomathamsanqa Majozi	Africa Health Research Institute	Public Engagement
Nceba Gqaleni	Africa Health Research Institute	Public Engagement
Hannah Keal	Africa Health Research Institute	Communications
Phumla Ngcobo	Africa Health Research Institute	Communications
Costa Criticos	Africa Health Research Institute	Operational Oversight
Raynold Zondo	Africa Health Research Institute	Operational Oversight
Dilip Kalyan	Africa Health Research Institute	Operational Oversight
Clive Mavimbela	Africa Health Research Institute	Operational Oversight
Anand Ramnanan	Africa Health Research Institute	Procurement
Sashin Harilall	Africa Health Research Institute	Grants Office
Kennedy Nyamande	University of KwaZulu-Natal	Pulmonology Consultant
Jaikrishna Kalideen	Perumal and Partners Radiologist Inc	Radiologist
Ramesh Jackpersad	Jacpersad and Partners Inc	Radiologist
Kgaugelo Moropane	Aurum Innova (Pty) Ltd	Radiographer
Boitsholo Mfolo	Aurum Innova (Pty) Ltd	Radiographer
Khabonina Malomane	Aurum Innova (Pty) Ltd	Radiographer
